# Supplementary material for: Structure-guided antisense oligonucleotides selectively modulate frameshifting of a human gene
Source: bioRxiv. 2026 Sep 7:2026.09.03.748613. Preprint. [Version 1] doi: 10.64898/2026.09.03.748613 (PMC13622457; doi:10.64898/2026.09.03.748613)
Supplement: Supplementary file 1 [file NIHPP2026.09.03.748613V1-supplement-1.pdf]

bioRxiv preprint doi: https://doi.org/10.64898/2026.09.03.748613; this version posted September 7, 2026. The copyright holder for this preprint (which was not certified by peer review) is the author/funder, who has granted bioRxiv a license to display the preprint in perpetuity. It is made available under a CC-BY 4.0 International license.

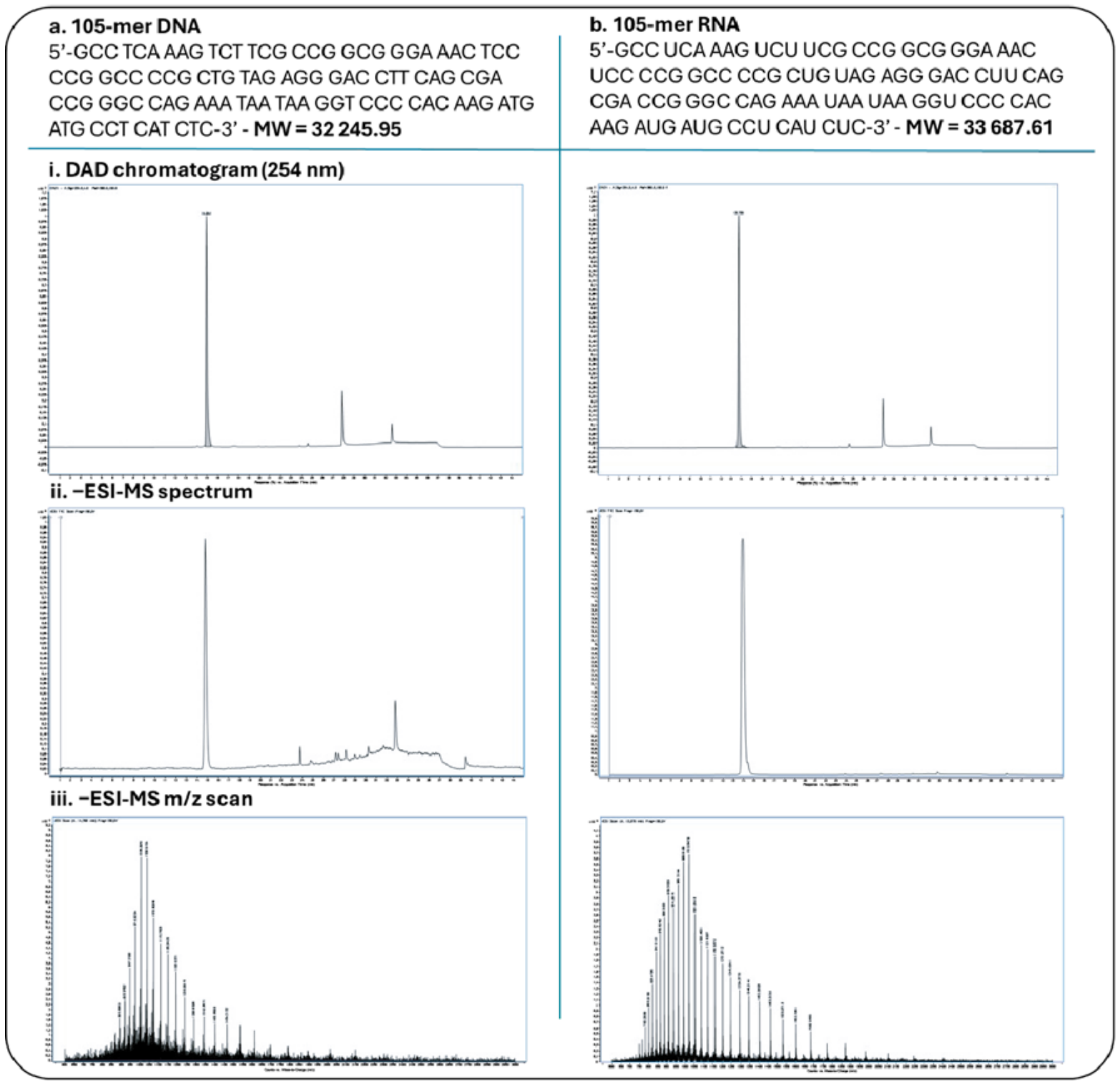

Supplementary Figure 1: Analytical characterization of the 105-nt synthetic model sequence mimicking the *PEG10* pseudoknot (a) Synthetic DNA version of the 105-nt model sequence. **(i)** Diode-array detector (DAD) chromatogram recorded at 254 nm. **(ii)** Negative-mode electrospray ionization mass spectrum (−ESI-MS). **(iii)** Deconvoluted −ESI-MS spectrum confirming the expected molecular mass. **(b)** Synthetic RNA version of the 105-nt model sequence. **(i)** DAD chromatogram at 254 nm. **(ii)** −ESI-MS spectrum. **(iii)** −ESI-MS m/z scan.

bioRxiv preprint doi: https://doi.org/10.64898/2026.09.03.748613; this version posted September 7, 2026. The copyright holder for this preprint (which was not certified by peer review) is the author/funder, who has granted bioRxiv a license to display the preprint in perpetuity. It is made available under a CC-BY 4.0 International license.

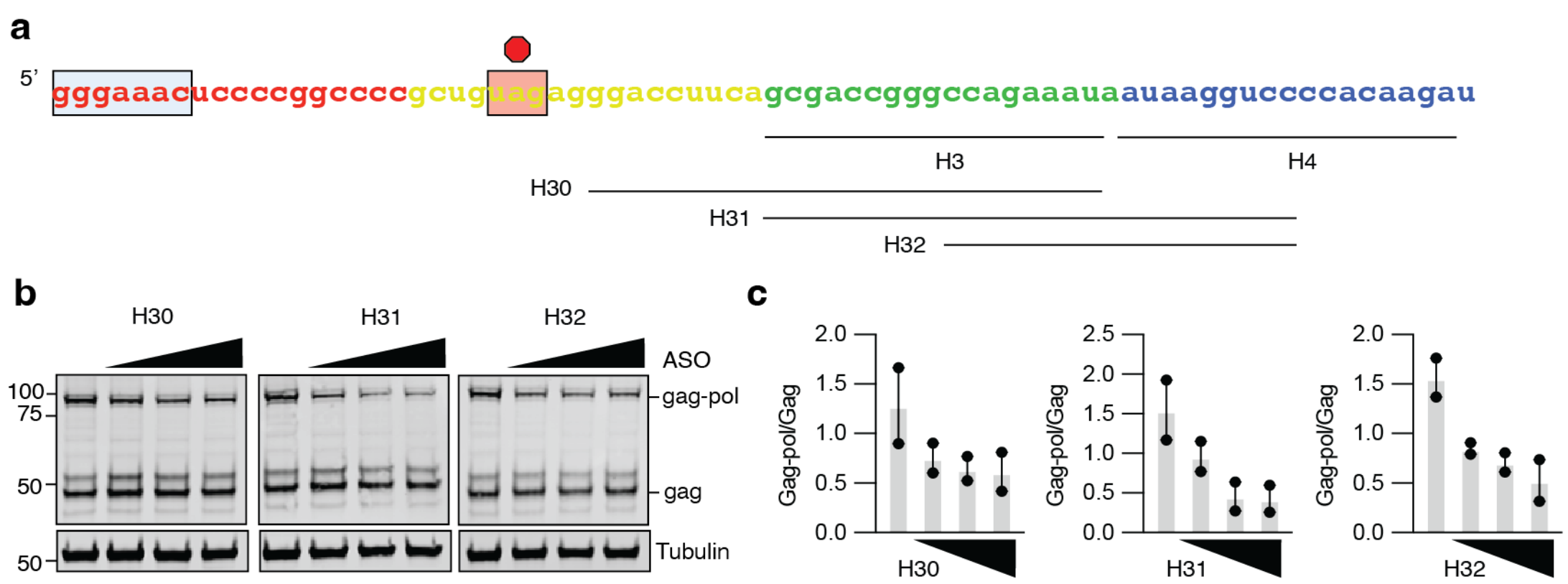

Supplementary Figure 2: Second-generation thiomorpholino ASOs are not meaningfully better than first-generation ASOs in modulating gag-pol:gag ratio. a) Schematic of second-generation human *PEG10*-targeting ASOs as compared to H3 and H4. H30 and H31 are longer ASOs; H32 is an 18-mer that spans regions of H3 and H4. **b)** Representative western blot comparing efficacy of second-generation ASOs to modulate PEG10 frameshifting. HEK293 cells were transfected with 0, 50, 100, or 200 nM ASO as in Figure 3 and harvested 72 hours later for western blot. Shown is one of two representative blots. **c)** Quantitation of the gag-pol:gag ratio for each of the three second-generation ASOs. Data are from two representative experiments.

bioRxiv preprint doi: https://doi.org/10.64898/2026.09.03.748613; this version posted September 7, 2026. The copyright holder for this preprint (which was not certified by peer review) is the author/funder, who has granted bioRxiv a license to display the preprint in perpetuity. It is made available under a CC-BY 4.0 International license.

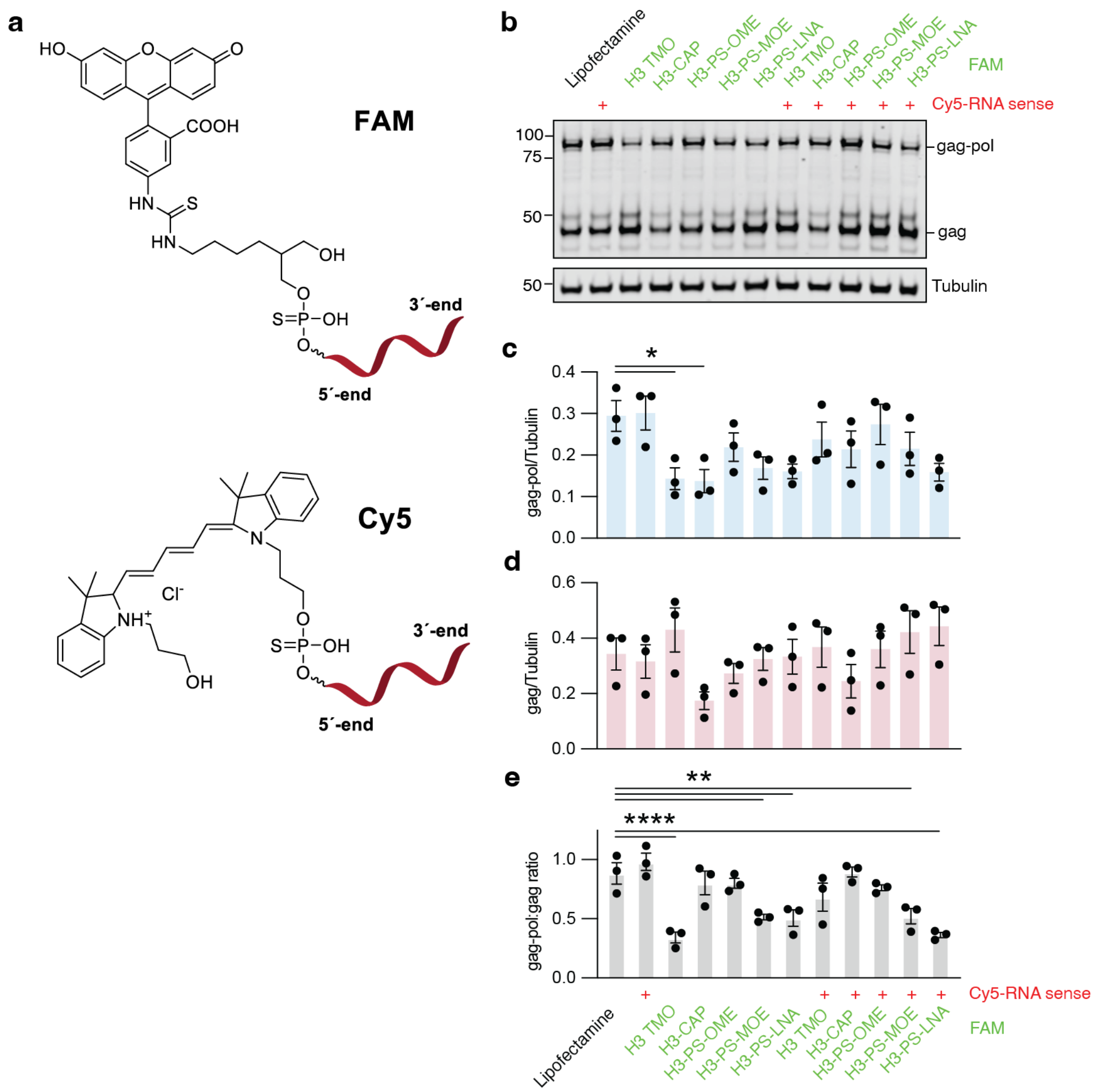

Supplementary Figure 3: Modification of ASOs with fluorophores results in similar effects on PEG10 frameshifting, but formation of RNA:ASO duplexes mitigates their effect. a) Schematic of fluorophore connection to ASO and RNA showing thiophosphate linkage between fluorophore and 5' end of oligonucleotide. **b)** Example western blot with FAM-labeled ASOs with and without pre-annealing to Cy5-labeled sense sequence of RNA 72 hours after transfection into HEK293 cells at 100 nM. Shown is one of three representative blots. **c-e)** Quantitation of gag-pol (c), gag (d), and the ratio of gag-pol:gag abundance (e) from (b). Statistics were determined by one-way ANOVA with multiple comparisons.

bioRxiv preprint doi: https://doi.org/10.64898/2026.09.03.748613; this version posted September 7, 2026. The copyright holder for this preprint (which was not certified by peer review) is the author/funder, who has granted bioRxiv a license to display the preprint in perpetuity. It is made available under a CC-BY 4.0 International license.

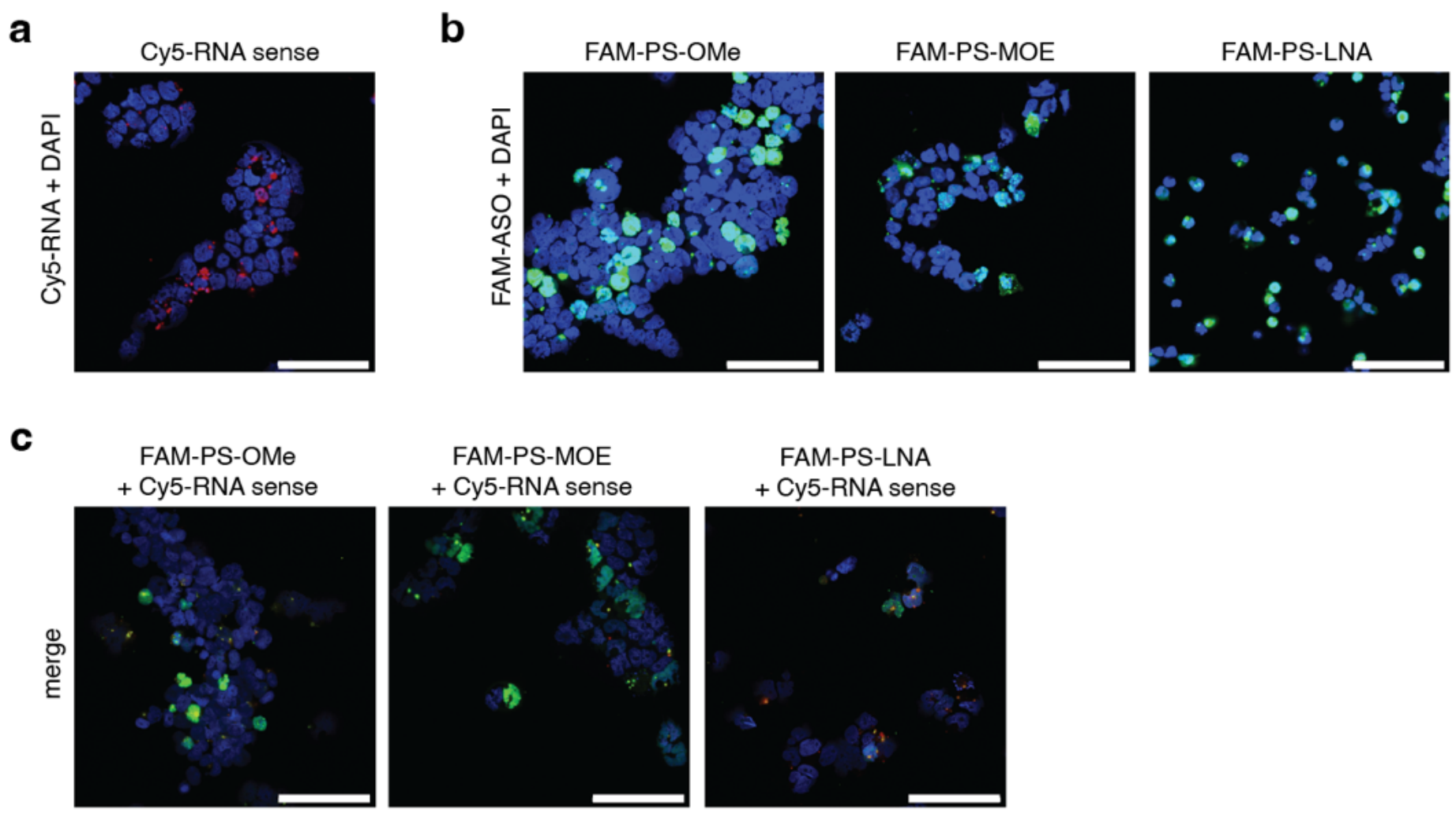

Supplementary Figure 4: Pre-annealing of OMe, MOE, and LNA ASOs with RNA changes their localization. a) Micrograph of HEK293 cells transfected with Cy5-labeled RNA. **b)** Micrograph of HEK293 cells transfected with FAM-labeled PS-OMe, PS-MOE, or PS-LNA. **c)** Micrograph of HEK293 cells transfected with pre-annealed mixtures of Cy5-labeled RNA with either FAM-labeled PS-OMe, PS-MOE, or PS-LNA. For (a-c), scale bar is 100 μm and images are representative of 3 experiments. Within each sub-panel, microscope and collection parameters were kept the same between ASO types.

bioRxiv preprint doi: https://doi.org/10.64898/2026.09.03.748613; this version posted September 7, 2026. The copyright holder for this preprint (which was not certified by peer review) is the author/funder, who has granted bioRxiv a license to display the preprint in perpetuity. It is made available under a CC-BY 4.0 International license.

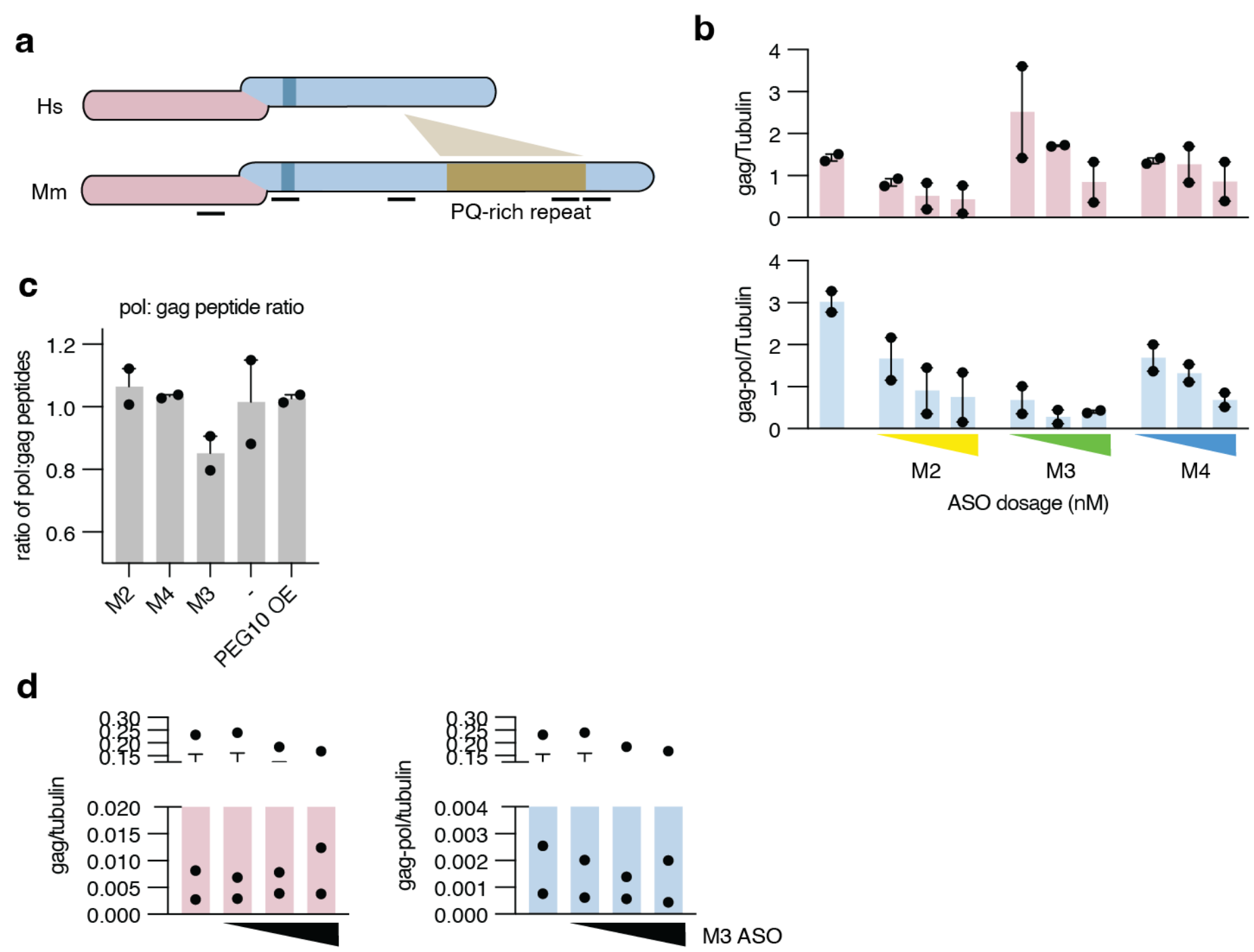

Supplementary Figure 5: Murine *Peg10* is similarly affected by Loop 2-targeting TMOs. a) Schematic showing the general amino acid sequence divergence between PEG10 in human and Peg10 in mouse. In the middle of the pol region of murine Peg10 is a proline and glutamine-rich (PQ-rich) repeat region. Black bars at the bottom represent the approximate locations of peptides that were identified by mass spectrometry of LLC cells in (d). **b)** Gag (top) and gag-pol (bottom) levels quantified for transfected 3T3 cells shown in Figure 7c. Statistics were performed via one-way ANOVA with multiple comparisons. **c)** Murine LLC cells were transfected with 100 nM ASO and harvested 48 hours later for mass spectrometry analysis of endogenous murine Peg10 protein. To trigger MS2 quantitation of Peg10, a sample was included with 5% lysate from cells overexpressing (OE) murine HA-tagged Peg10 protein. Peptide abundances for each sample's identified Peg10 peptides were first compared to the average of all samples. Each peptide was assigned to gag or pol regions. Then, those averages were used to generate a pol:gag ratio for each sample. Data are from two independent transfections. **d)** Gag (top) and gag-pol (bottom) levels quantified for transfected murine cortical neurons shown in Figure 7d. Statistics were performed via one-way ANOVA with multiple comparisons. Gag and pol levels are shown with two-piece y-axis because of variability of Peg10 signal across experiments.
